# A Review of Foods of Plant Origin as Sources of Vitamins with Proven Activity in Oxidative Stress Prevention according to EFSA Scientific Evidence

**DOI:** 10.3390/molecules28217269

**Published:** 2023-10-25

**Authors:** María Ciudad-Mulero, Laura Domínguez, Patricia Morales, Virginia Fernández-Ruiz, Montaña Cámara

**Affiliations:** Department of Nutrition and Food Science, Faculty of Pharmacy, Complutense University of Madrid, Pza Ramón y Cajal, s/n, E-28040 Madrid, Spain; mariaciudad@ucm.es (M.C.-M.); ladoming@ucm.es (L.D.); patricia.morales@farm.ucm.es (P.M.); mcamara@ucm.es (M.C.)

**Keywords:** vitamins, health, antioxidant capacity, oxidative stress, foods of plant origin, natural sources

## Abstract

Beyond their nutritional benefits, vitamins could decrease the risk of chronic diseases due to their potent antioxidant capacity. The present work is aimed at reviewing the state of the art regarding (1) the vitamins involved in oxidative stress prevention in accordance with the requirements established by the European Food Safety Authority (EFSA) and (2) the foods of plant origin that are sources of those vitamins and have potential benefits against oxidative stress in humans. According to the European regulations based on EFSA scientific evidence, riboflavin, vitamin C, and vitamin E are those vitamins subjected to the approved health claim “contribute to the protection of cells from oxidative stress”. Scientific studies conducted in humans with some natural food sources of riboflavin (almonds, wheat germ, mushrooms, oat bran), vitamin C (guava, kale, black currant, Brussels sprouts, broccoli, orange), and vitamin E (hazelnuts, almonds, peanuts, pistachio nuts, extra virgin olive oil, dates, rye) have been performed and published in the literature. However, no food of plant origin has obtained a favorable EFSA opinion to substantiate the approval of health claims related to its potential properties related to oxidative stress prevention. Further studies (concretely, well-controlled human intervention studies) must be carried out in accordance with EFSA requirements to provide the highest level of scientific evidence that could demonstrate the potential relationship between foods of plant origin and antioxidant capacity. This review could be useful for the scientific community to study the application of health claims referring to the antioxidant capacity potentially exerted by foods of plant origin.

## 1. Introduction

Oxygen is considered a vital source of energy necessary to sustain life in living organisms [1]. Oxygen is the driving force not only for the development, growth, metabolism, and reproduction functions but also for the maintenance of a great number of cellular processes in the human organism [2,3]. Paradoxically, oxygen is an indirect liability for some negative effects [4,5] as it can easily give rise to unstable oxygen molecules known as “Reactive Oxygen Species” (ROS) [6,7,8].

ROS include hydrogen peroxide (H_2_O_2_), hydroxyl radicals (OH), and the superoxide anion (O_2_^−^), which inherently possess chemical properties that lend high reactivity to biological targets [9]. Under physiological situations, the ‘homeostasis’ or the balance between free radical production and antioxidant mechanisms exists so that the organism’s functioning is adequate and human health is preserved [10,11]. In pathological and stress conditions, defense systems are overcome by reactive oxygen molecules. This phenomenon is commonly known as “oxidative stress”, which can be defined as “a state in which oxidation exceeds the antioxidant systems in the body secondary to a loss of the balance between” [12,13]. ROS are capable of interacting with a great number of biomolecules in the organism (e.g., amino acids, proteins, nucleic acids, lipids, etc.) and provoke cellular damage, which in turn causes the disruption and dysfunction of essential cellular processes and, finally, apoptosis [14,15,16]. Hence, oxidative stress is considered a major risk factor for the development of several pathologies including cancer, cardiovascular diseases, and neurodegenerative disorders, among others [16,17,18,19,20,21].

Epidemiologic studies have strongly demonstrated an inverse association between the regular consumption of foods of plant origin, mainly fruits and vegetables, and the prevalence of diseases. This protective role has been attributed to the presence of components with antioxidant activity [22]. Dietary antioxidants include vitamins (vitamin C, vitamin E), carotenoids (carotenes and xanthophylls), and phenolic compounds, also called polyphenols (flavonoids, phenolic acids, lignans and stilbenes) [10,23]. In addition, there are other compounds found in food that also have antioxidant properties, such as minerals and some constituents of dietary fiber [10].

Within micronutrients, vitamins are necessary for energy production and the supporting of body structures. There are two principal groups, based on their solubility: fat-soluble vitamins, such as vitamins A, D, E, and K; and water-soluble vitamins (B, C). It is well known that vitamins from both categories can simultaneously act in an organism in a synergistic action. A large majority of vitamins are precursors to certain coenzymes whose mechanism of action mainly lies in the enzymatic regulation of metabolism. Beyond their nutritional benefits and the prevention of deficiency illnesses, vitamins have been demonstrated to decrease the risk of some disorders and chronic diseases due to their potent antioxidant capacity [24,25,26].

In Europe, the European Food Safety Authority (EFSA) is the responsible for assessing the last scientific evidence regarding the potential health benefits attributed to specific foods and/or food constituents to support the authorization and approval of health claims by the European Commission (EC) [27]. According to Regulation (EC) No 1924/2006 of the European Parliament and of the Council of 20 December 2006, health claims are considered those messages included in the labeling, presentation, and/or advertising of food products, that state, suggest, or imply that the consumption of a specific food item or the intake of one of its constituents exerts a beneficial effect on human health. In addition, claims must be based on the highest scientific evidence to establish an ethical commitment with consumers that will avoid any misunderstanding regarding the nutritional adequacy of other food products. In fact, health claims must be accompanied by a statement informing consumers that the food product in question must be one more component of a balanced and varied diet to avoid its excessive consumption [28].

The present work is aimed at reviewing the state of the art regarding (1) the vitamins with proven protective effect against oxidative stress in accordance with the requirements established by the EFSA and (2) the foods of plant origin that are sources of those vitamins and have potential benefits against oxidative stress in humans.

## 2. Results and Discussion

### 2.1. Vitamins with EFSA Scientific Evidence Related to the Oxidative Stress Prevention

As it was mentioned in the introduction, the approval of health claims in Europe is a comprehensive and complex process in which the EFSA makes a scientific judgment on whether one specific claimed effect should be authorized or not. To obtain a favorable EFSA opinion, it must meet specific requirements. For instance, the food or constituent subject to the claim must have demonstrated a beneficial physiological effect, which must be sufficiently defined. A complete scientific dossier must be provided to the EFSA in order to demonstrate the potential relationship between the food/constituent and the physiological effect in question [29,30,31].

Well-controlled human studies are fundamental to scientifically support health claims, with intervention studies being the ones that provide the strongest scientific evidence. In the case of observational studies, the control of other factors different from the food/constituent that could influence the results are of great importance. Unpublished human studies can be considered as long as detailed descriptions of the methodologies performed in those studies are provided in the scientific dossier. Animal studies are useful to partially support the substantiation of health claims if they both clearly indicate the potential mechanisms of action as well as demonstrate the biological plausibility of their claims. In any event, all studies selected to be included in the scientific dossier to obtain a favorable EFSA opinion should be of high quality in terms of reporting and methodology [30].

The study design should be appropriate in terms of (1) the pattern of consumption and quantity of the food/constituent in question, (2) the study group, which should be representative to extrapolate the findings to the general population, and (3) the outcome measures, which should be in accordance with rigorous methodological procedures included in generally accepted guidelines published by renowned institutions and societies in that area. The claimed effect must be consistent between studies as well [29,31].

To date, only three vitamins, riboflavin (vitamin B_2_), vitamin C, and vitamin E, have clearly demonstrated significant antioxidant properties against oxidative stress according to EFSA considerations and requirements [32].

Scientific evidence that substantiated the antioxidant capacity of these vitamins was summarized in four scientific opinions published by the EFSA Panel on Dietetic Products, Nutrition and Allergies (NDA), and two of them focused on vitamin E [30,33,34,35]. In these reports, it was indicated that riboflavin, vitamin C, and vitamin E are recognized nutrients sufficiently characterized that can be adequately measurable in food products by established official methods, and they are allowed to be used in foods and food supplements in accordance with Regulation (EC) No 1925/2006, on the addition of vitamins and minerals and of certain other substances to foods [36], and Directive 2002/46/EC, on the approximation of the laws of the Member States relating to food supplements [37]. The beneficial physiological effect was the protection of DNA, proteins, and lipids from oxidative stress, and the general population was considered the target study group [30,33,34]. The EFSA affirmed that riboflavin has a protective role against lipid peroxidation due to its implication in the glutathione redox cycle. In addition, it was also reported that riboflavin deficiency is associated with increased lipid peroxidation [34]. Regarding vitamin C, the EFSA indicated that it takes part in the antioxidant defense system, a complex network with mutual interactions and synergetic effects among different endogenous and exogenous components. In vivo studies demonstrated that vitamin C (ascorbate form) can be considered a potent scavenger of reactive oxygen molecules in different cells, structures, tissues, and/or organs (e.g., activated leukocytes; lung and gastric mucosa) and that it is able to attenuate lipid peroxidation [33]. The EFSA established that the role of vitamin E in the protection of the biomolecules previously mentioned can be applied to all ages, including infants and young children (from birth to 3 years of age) [35]. Moreover, it is reported that vitamin E can act as a chain-breaking antioxidant by avoiding the propagation of lipid peroxidation [30,35].

With all these considerations, the EFSA Panel concluded that sufficient scientific studies had been provided to evaluate and prove a cause–effect relationship between the dietary intakes of riboflavin, vitamin C, and vitamin E and the protection of DNA, proteins, and lipids from damage caused by oxidative stress.

The health claim approved for riboflavin, vitamin C, and vitamin E is related to the protection of DNA, proteins, and lipids from oxidative damage, and it uses the following wording: “Contribute to the protection of cells from oxidative stress”. Direct measurements of oxidative damage to these biomolecules, which is necessary to support this claim, can be obtained in vivo using specific methodologies and procedures included in the corresponding EFSA guidance [31].

**Figure 1:**
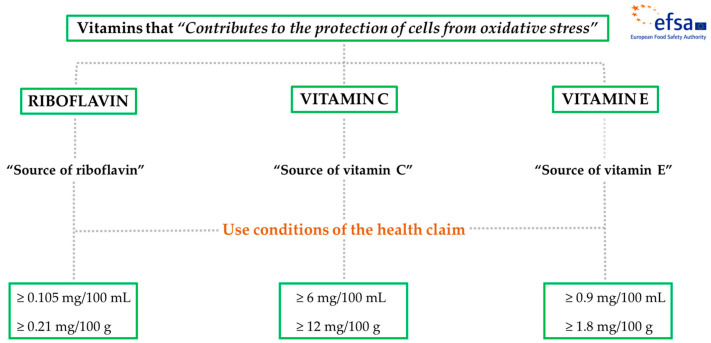
Overview of the application of use conditions to the health claim “Contributes to the protection of cells from oxidative stress” related to riboflavin, vitamin C, and vitamin E [28,38,39].

According to Regulation (EC) No 1924/2006; Regulation (EU) 432/2012, establishing a list of permitted health claims made on foods other than those referring to the reduction of disease risk and to children’s development and health; and Regulation (EU) No 1169/2011, on the provision of food information to consumers, those food products intended to use the above-mentioned health claim in their labeling, presentation, and/or advertisement must meet the specific requirement of being at least a “Source of” these vitamins. That is, significant contents of the Nutrient Reference Value (NRV) of riboflavin, vitamin C, and vitamin E. NRV of vitamins are included in Annex XIII of Regulation (EU) No 1169/2011. Depending on the type of food product, a significant content will mean 15% NRV (foods) or 7.5% NRV (beverages) supplied by 100 g or 100 mL of product, respectively [28,38,39]. Table 1 and Figure 1 summarize the conditions required to use this health claim.

The EFSA Panel has evaluated the potential relationship between the consumption of some foods of plant origin and the protection of DNA, proteins, and lipids from oxidative damage. Examples of the foods of plant origin that were subject to EFSA evaluation were the following: persimmon fruit (*Diospyros kaki*), banana (*Musa* L.), guava (*Psidium* L.), lemon (*Citrus limonium*), grapefruit (*Citrus paradisi*), grapes (*Vitis vinifera*), prunes (dried plums), cherries (*Prunus cerasus*), cranberry (*Vaccinium macrocarpon*), blackberry (*Morus nigra*), bilberry (*Vaccinium myrtillus*), wolfberry (*Lycium barbarum*), pitanga (*Eugenia uniflora*), artichoke (*Cynara scolymus*), onion (*Allium cepa*), acerola (*Malphigia* L.), wheat sprouts, purple grape juice, black currant juice, and seed oils from wild berries (e.g., lingonberry, bilberry, and cranberry) and from domestic berries (e.g., strawberry, and raspberry). In vitro, in vivo, and ex vivo studies assessing the capacity to scavenge reactive oxygen molecules and to delay or avoid DNA, protein, and lipid oxidation were carried out with the foods of plant origin previously mentioned. No human studies were performed and included in the dossier. The EFSA Panel concluded that scientific evidence from animal studies is insufficient to extrapolate the results obtained to humans as required by Regulation (EC) No 1924/2006, and so, it could not prove a cause–effect relationship between the consumption of these foods of plant origin and the protection of DNA, proteins, and lipids from damage caused by oxidative stress [34].

Although no food of plant origin has obtained a favorable EFSA opinion, numerous studies carried out in humans with natural food sources of vitamins with EFSA scientific evidence related to oxidative stress prevention (riboflavin, vitamin C, and vitamin E) have been performed and published in the literature. These studies are described in the next section, and they could be a good starting point to substantiate health claims referring to the antioxidant capacity potentially exerted by foods of plant origin.

### 2.2. Foods of Plant Origin as Sources of Vitamins with Scientific Evidence Related to Oxidative Stress Prevention

As is mentioned in Section 3 (Materials and Methods), foods of plant origin subjected to this review met the condition “Source of riboflavin”, “Source of vitamin C” or “Source of vitamin E” according to the European Regulation in force. A total of 30 foods of plant origin (ten foods for each vitamin) with the highest contents of these vitamins were selected in accordance with data published in the Official Databases of food composition [40,41,42].

#### 2.2.1. Riboflavin

Riboflavin, also called vitamin B_2_, is an hydrosoluble vitamin implicated in a wide range of biological processes as it acts as a coenzyme for several flavoprotein enzymes, either in the form of flavin mononucleotide (FMN) or in the form of flavin adenine dinucleotide (FAD). Riboflavin catalyzes both oxidation and reduction reactions as well as electron transport. This vitamin is involved in redox reactions in several metabolic pathways, and it plays a key role in protein and energetic metabolism, being involved in the biosynthesis and catabolism of carbohydrates, fatty acids, and amino acids. It is also implicated in the metabolism of other vitamins of group B, such as pyridoxin (vitamin B_6_), folate (vitamin B_9_), and cyanocobalamin (vitamin B_12_) [43].

The Daily Reference Intake (DRI) for riboflavin is 1.4 mg/day [38], and the deficiency of this vitamin is usually caused by an inadequate diet. Riboflavin deficiency is associated with low growth in children, impaired iron absorption, cardiovascular diseases, and peripheral neuropathy [44,45]. In contrast, it has been suggested that the higher dietary intake of riboflavin could be associated with protective effects against aging or certain pathologies such as cancer [45].

The main dietary sources of this vitamin are meats, dairy products, fatty fish, legumes, liver, nuts, and eggs, as well as some vegetables and fruits [34,44,46,47]. Table 2 includes the ten foods of plant origin with the highest content of riboflavin according to Official Databases of food composition [40,41,42]. All meet the requirements of “Source of riboflavin” (≥0.21 mg riboflavin/100 g product) established in the European regulation in force. Thus, these foods would be able to use the following approved health claim: “Riboflavin contributes to the protection of cells from oxidative stress” [28,38,39]. Specifically, lupin also contains some compounds considered as antinutrients, such as phytic acid, among others. This compound reduces the bioavailability of minerals by forming complexes with them, but phytic acid could also act as an antioxidant agent due to its ability to inhibit the formation of the iron-catalyzed hydroxyl radical [10,48].

Among the foods included in Table 2, the roles against oxidative stress of almonds, wheat germ, mushrooms, and oat bran have been studied and evaluated in human clinical trials.

Mohammadi et al. (2020) performed a randomized, double-blind, placebo-controlled trial to assess the potential beneficial effects of wheat germ consumption on oxidative stress status and metabolic markers. A total of 80 type II Diabetes Mellitus patients were selected and randomly grouped in two clusters: a wheat germ group, consisting of patients with a daily consumption of 20 g of wheat germ for 12 weeks, and a placebo group. At the end of the intervention, malondialdehyde (MDA) levels, antioxidant capacity, serum lipid profiles, and glycaemic indices were evaluated. The authors concluded that those patients subjected to wheat germ consumption reported lower total cholesterol levels and higher antioxidant capacity [49].

Jenkins et al. (2008) suggested that almonds have interesting antioxidant potential. Their results demonstrated that the dietary supplementation of riboflavin (73 g/day) during 4 weeks in older hyperlipidemic patients was associated with reductions in different markers of lipid peroxidation, including the serum concentrations of malondialdehyde (MDA), and urinary isoprostanes [50]. Liu et al. (2013) performed a randomized crossover, controlled feeding trial in which a specific quantity of almonds (56 g/day, approximately) was incorporated into a healthy diet in Chinese patients with type 2 Diabetes Mellitus. These authors observed that adherence to an ‘almond diet’ significantly decreased the values of circulating oxidized LDL in patients in comparison with the control so that the consumption of these nuts could ameliorate the oxidative stress within the context of a balanced and varied diet [51]. Recently, Rakic et al. (2022) carried out a single-blinded, randomized–controlled trial to investigate the effects of daily almond consumption on cognition in healthy middle-aged/older adults (50–75 years). In this study, oxidative status was assessed at baseline at three and six months during the intervention, and authors reported that serum markers of oxidative stress were not significantly different throughout the study among the groups [52].

Mushrooms are foods of plant origin that constitute an important source of riboflavin and whose role against oxidative stress has been investigated as well. Poddar et al. (2013) conducted, for 12 months, a randomized clinical trial, enrolling 73 obese adults, in order to examine the effect of substituting red meat with mushrooms on weight loss and maintenance. These authors also studied the influence of this dietary intervention on different health parameters including lipid profile and biochemical indicators of oxidative stress, among others. In accordance with the results, dietary interventions consisting of substituting red meat with mushrooms were linked to both a loss of weight as well as an improvement in the body’s composition. In addition, it was observed that there were positive changes in several health indicators, such as blood pressure, cholesterol (total, LDL, and HDL), and in markers of inflammation. However, oxidized LDL, which is an indicator of oxidative stress, did not improve in the study previously mentioned [53].

Sahrir et al. (2017) evaluated the potential effects of oat bran on lipid profile and antioxidant parameters in 47 young men (university students aged 20 years old). Those participants who were randomly assigned to the intervention group were asked to daily consume 18 g of oat bran for 8 weeks. Intervention with oat bran was performed using powder supplements. The results revealed that the daily consumption of oat bran was able to both reduce total cholesterol and low-density lipoprotein cholesterol concentrations as well as increase superoxide dismutase activity [54].

#### 2.2.2. Vitamin C

Vitamin C (L-ascorbic acid) is an hydrosoluble organic compound that is necessary for the normal metabolic functioning of the human body. Vitamin C is an important component of the diet as humans do not have the ability to synthesize it via the glucuronic acid pathway. This vitamin exhibits a high reducing power, giving it remarkable antioxidant properties. Vitamin C participates as a cofactor in a great number of biochemical reactions including the synthesis of collagen, catecholamines, and carnitine. Vitamin C also participates in the metabolism of cholesterol [55,56].

The Daily Reference Intake (DRI) for vitamin C is 80 mg/day [38], and an inadequate diet could cause vitamin C deficiency, which is associated with increased all-cause mortality [57]. In addition, the prolonged severe deficiency of vitamin C results in a clinical syndrome named scurvy, which can cause human death if it is not appropriately treated. In addition to dietary factors, socioeconomic, environmental, and demographic factors also influence vitamin C status [58].

The main dietary sources of this essential vitamin are fruits (orange, pomelo, kiwifruit, strawberries, cranberries, mangoes, papayas, melons, etc.) and vegetables (broccoli, cauliflower, cabbage, cantaloupe, spinach, asparagus, Brussels sprouts, etc.). According to the scientific literature, the impact of fruit intake on plasma levels of vitamin C is potentially stronger than the effect of vegetable intake [56,58,59]. Table 3 includes the ten foods of plant origin with the highest concentrations of vitamin C in accordance with the Official Databases of food composition [40,41,42]. All meet the requirements of “Source of vitamin C” (≥12 mg vitamin C/100 g product) established in the European regulation in force. Thus, these foods would be capable of using the following authorized health claim: “Vitamin C contributes to the protection of cells from oxidative stress” [28,38,39].

Among the foods included in Table 3, the roles against oxidative stress of guava, kale, black currant, Brussels sprouts, broccoli, and orange have been studied and evaluated in human clinical trials. It is important to highlight that vitamin C is thermolabile and the contents shown in Table 3 for Brussels sprouts, red pepper, broccoli, and cauliflower could be decreased via heat treatment (boiling, cooking, etc.).

The effects of guava consumption on total antioxidant status and lipid profile were assessed by Rahmat et al. (2004, 2006). In both clinical trials, healthy young men (18–24 years old) were recruited and asked to consume guava (approximately 400 g of fruit/day) for 4 weeks. Blood samples were collected at the end of the intervention, and the activity levels of antioxidant enzymes (glutathione peroxidase, glutathione reductase), as well as lipid markers such as total cholesterol, triglycerides, LDL cholesterol, and HDL-cholesterol, were measured. The results revealed a decrease in oxidative stress due to a significant increase in the antioxidant status during the period of guava consumption. Plasma levels of total cholesterol and triglycerides were slightly higher in these individuals; however, HDL-cholesterol concentrations also increased and were associated with lower risks of heart attack and cardiovascular disease [60,61].

Lee et al. (2018) investigated the potential antioxidant capacity of kale juice. The authors randomly selected 84 subclinical hypertensive patients who daily consumed 300 mL of kale juice for 6 weeks. After the intervention, DNA damage caused by oxidative stress was reduced, and catalase and glutathione peroxidase activities in erythrocytes increased. According to the authors, this fact could be explained due to the higher plasma vitamin C concentrations reported in these individuals [62].

A randomized controlled trial carried out by Khan et al. (2014) evaluated the beneficial effects of the consumption of a black currant juice drink rich in vitamin C and other bioactive compounds (e.g., polyphenols and anthocyanins) on oxidative stress. A total of 66 healthy participants were selected and allocated in three groups: the placebo group, in which individuals consumed flavored water, and two groups corresponding to low juice consumption (6.4% juice, final diluted concentration of 1.1 mg vitamin C/100 mL, 27.3 mg total polyphenols/100 mL, and 4 mg anthocyanins/100 mL) and high consumption (20% juice, final diluted concentration of 10.2 mg vitamin C/100 mL, 81.5 mg total polyphenols/100 mL, 14.3 mg anthocyanins/100 mL). All participants consumed 250 mL of the black currant juice drink four times per day for 6 weeks, which meant that the participants recruited in the low juice consumption group ingested 11 mg vitamin C/day, 273 mg total polyphenols/day, and 40 mg anthocyanins/day while the individuals in the high juice consumption group received 102 mg vitamin C/day, 815 mg total polyphenols/day, and 143 mg anthocyanins/day. All participants were requested to follow their normal diets and exercise routines without modifications except for the consumption of the juice drink. Measurements of both one marker of oxidative stress (F2-isoprostanes) as well as plasma concentrations of vitamin C were performed in all groups. According to the results obtained, participants who had had high consumption levels of the black currant juice drink showed higher plasma levels of vitamin C and lower F2-isoprostane concentrations in comparison with the low consumption and placebo groups. It can be concluded that the consumption of this juice rich in vitamin C and other compounds (polyphenols and anthocyanins) could ameliorate oxidative stress [63].

According to the scientific literature, Brussels sprouts are other foods of plant origin with beneficial effects on antioxidant status. Various clinical trials have been performed in humans to study the influence of the daily consumption of Brussels sprouts on the levels of an antioxidant enzyme, glutathione S-transferase. Bogaards et al. (1994) and Nijhoff et al. (1995) reported higher plasma concentrations of glutathione S-transferase in men but not in women. This finding could be explained due to sex differences on induction of this enzyme following Brussels sprouts consumption [64,65,66]. In recent years, a human intervention study carried out by Hoelzl et al. (2007) evaluated the protective effect against oxidative DNA damage due to the consumption of Brussels sprouts. After consuming 300 g of these vegetables per day, serum vitamin C concentrations were increased by 37%, and DNA damage caused by hydrogen peroxide significantly decreased (39%). However, there was no direct correlation between higher concentrations of vitamin C and lower levels of oxidative DNA damage. Further studies are needed to elucidate the influence of the vitamin C content in Brussels sprouts on oxidative stress [67].

Riso et al. (2009, 2010, 2014) conducted three clinical trials with the aim of assessing the antioxidant properties of broccoli, an important source of vitamin C. The authors measured the potential influence of the regular consumption of this vegetable on biomarkers of DNA damage and glutathione S-transferase (GST) activity, a key component of the endogenous defense system of the organism against oxidative stress. The authors concluded that broccoli consumption could significantly improve antioxidant status in participants by reducing oxidized DNA lesions by 41% and increasing the resistance of DNA strands against H_2_O_2_-induced breaks by 23%, with a 95% confidence interval. Broccoli intervention did not influence GST activity. More studies focusing on the positive effects of broccoli on the endogenous defense system are necessary to confirm these findings [68,69,70].

As shown in Table 3, orange, which is considered a source of vitamin C, has a remarkable antioxidant capacity, and it plays an important role against oxidative stress. Different authors have focused their research on the study of antioxidant effects of orange juice [71,72,73,74]. Riso et al., (2005) performed a human intervention study with blood orange juice in order to test in vivo its potential protection against oxidative stress. In the crossover study previously mentioned, 60 healthy females were enrolled and allocated in two groups. On the one hand, the participants in the first group followed a standard diet without orange juice for 21 days, and after this period, they consumed 600 mL/day of blood orange juice for 21 days. On the other hand, the participants in the second group followed the opposite pattern: during the first 21 days, they consumed 600 mL/day of blood orange juice, and during the next 21 days, they followed a standard diet without orange juice. The blood orange juice tested in this study contained high amounts of vitamin C, anthocyanins, and carotenoids, and the authors quantified plasma levels of vitamin C, cyanidin-3-glucoside, and carotenoids before and after each intervention period. In addition, several biomarkers of oxidative stress were evaluated. It was observed that the consumption of blood orange juice was associated with both higher values of plasma vitamin C as well as with increased plasmatic levels of cyanidin-3-glucoside, β-cryptoxanthin, and zeaxanthin. It was also reported that lymphocyte DNA resistance to oxidative stress improved because of this intervention. These results suggest that blood orange juice seems to improve the antioxidant defense systems, but further studies are necessary to assess the long-term effects of its regularly intake [75].

In the case of orange juice, Guarnieri et al. (2007) and Constans et al. (2015) observed that the antioxidant effect related to the consumption of orange juice is explained not only by the presence of vitamin C but also by the content of other phytochemicals such as hesperidin, among others [72,73]. These findings were found in an interesting randomized, single-blinded, crossover trial performed to investigate the potential impact of blood orange juice on antioxidant markers in comparison with a control beverage, which mimicked the orange juice composition (mainly vitamin C, but no phytochemicals). In addition, other parameters like cardiovascular factors and endothelial function were also evaluated. The study included 25 male volunteers with two factors of cardiovascular risk. For 4 weeks, participants received 200 mL (3 times daily) of blood orange juice or a control beverage. The results showed that the daily consumption of orange juice could contribute to the improvement of some antioxidant parameters, providing a beneficial health effect. This positive effect was correlated to the hesperetin plasma levels. Therefore, the antioxidant properties of blood orange juice were related not only to the vitamin C content but also to the phytochemical composition of the food matrix [72].

Recently, Cara et al. (2022) carried out the first systematic review and meta-analysis to evaluate the effect of 100% orange juice interventions on common markers of oxidative stress and inflammation in both healthy adult populations as well as in subjects at risk for chronic diseases. These authors concluded that in general, interventions with 100% orange juice were related to beneficial or null effects on numerous markers of oxidative stress and inflammation in the selected individuals. However, they highlighted the need for further clinical trials to investigate the long-term effects derived from the consumption of 100% orange juice [71].

#### 2.2.3. Vitamin E

The term “vitamin E” is usually referred to a family of eight chemically related liposoluble compounds, namely tocopherols (α-, β-, δ- and γ- tocopherol) and tocotrienols (α-, β-, δ- and γ- tocotrienol). Regarding their chemical structure, each of these compounds is constituted by a chromanol head and an isoprene side chain. However, the saturation of the side chain is what differentiates tocopherols from tocotrienols, as this side chain is saturated in tocopherols while it has three double bonds in the case of tocotrienols, located between positions 3, 7, and 11. Tocopherols and tocotrienols can penetrate through biological membranes since their side chains are hydrophobic. Furthermore, the chromanol ring has a hydroxyl group (–OH) that can donate a hydrogen atom to reduce free radicals. Among all isoforms of vitamin E, it is known that α-tocopherol exhibits the highest biological activity [10,76,77].

The Daily Reference Intake (DRI) for vitamin E is 12 mg/day [38], and its deficiency, which is extremely unusual in humans, is caused by alterations in dietary fat absorption or metabolism, not by a diet consisting of low vitamin E intake [78].

The main dietary sources of vitamin E are nuts and vegetable oils such as extra virgin olive oil. In addition, it can also be found in smaller concentrations in some fruits and vegetables [79]. Table 4 includes the ten foods of plant origin with the highest contents of vitamin E according to the Official Databases of food composition (BEDCA, FRIDA, USDA). All meet the requirements of “Source of vitamin E” (≥1.8 mg vitamin E/100 g product) established in the European regulation in force. Hence, these foods could make use of the following health claim: “Vitamin E contributes to the protection of cells from oxidative stress” [28,38,39].

Most of the foods included in Table 4 have high caloric values but appropriate lipid profiles. Moreover, it is important to point out that a standard portion of nuts weighs less than 100 g, particularly 30 g. Among these foods (Table 4), the roles against oxidative stress of nuts (hazelnuts, almonds, peanuts, pistachio nuts), extra virgin olive oil, dates, and rye have been studied and evaluated in human clinical trials.

Postprandial oxidative stress is related to the increased susceptibility of an organism towards oxidative damage after the consumption of a meal rich in fat and/or carbohydrates. Di Renzo et al. (2017) conducted a randomized crossover trial in healthy humans to explore the antiatherogenic effects of the intake of 40 g of hazelnuts, evaluating the postprandial plasma oxidized LDL (ox-LDL) level after the consumption of a high-fat meal. These authors concluded that the intake of hazelnut reduced the plasmatic postprandial levels of ox-LDL so that it could be effective in the reduction of the risk of atherosclerosis. Moreover, they observed a reduction of oxidative and inflammatory gene expression as a consequence of the dietary intervention. However, studies on larger population are necessary before establishing solid conclusions [80]. In 2019, Di Renzo et al. studied the effect of the daily intake of hazelnuts on body composition and on genomic response in different genes related to oxidative stress and inflammation. The authors conducted a prospective clinical trial, enrolling 24 healthy volunteers who daily consumed a snack consisted of 40 g of hazelnuts for 6 weeks. After the intervention period, it was observed that hazelnut consumption was not associated with weight gain due to the potential improvement of each organism’s antioxidant capacity by upregulating genes involved in oxidant processes and inflammation [81].

The potential beneficial effects on oxidative biomarkers derived from almond consumption have also been studied. In a clinical trial designed by Jenkins et al. (2006), a total of 50 healthy humans were selected to investigate the reduction of postprandial glycemia, insulinemia, and oxidative damage. Individuals were subjected to four test meals; one of them consisted of the daily consumption of 60 g of raw unblanched almonds (California almonds) with white bread. Blood glucose and serum insulin levels, as well as protein thiol concentration (a marker of protein oxidative damage), were measured. The results showed that almond intake reduced the glycemic and insulinemic responses to bread and increased serum protein thiol concentration, decreasing the risk of oxidative damage to proteins [82]. Jenkins et al. (2008) performed a clinical trial with 27 hyperlipidemic subjects to study the reduction of lipid peroxidation through the consumption of whole almonds taken as snacks. Participants were grouped in three clusters: control group, intervention group supplemented with a full dose of almonds (73 g/day), and intervention group with a half dose of almonds. At the end of the intervention (4 weeks), it was observed that supplementation with a full dose of almonds could attenuate two biomarkers of lipid peroxidation: serum concentrations of malondialdehyde (MDA) and urinary isoprostanes. These results support that almonds have an important antioxidant capacity by reducing the oxidation of low-density lipoprotein cholesterol (LDL-c) [50]. Recently, Jung et al. (2018) carried out a randomized crossover clinical trial focused on the effect of almonds on vitamin E status and cardiovascular risk factors in 84 overweight/obese adults. In a period of 4 weeks, individuals consumed their habitual diets supplemented with either 56 g of almonds/day or isocaloric cookies. The intervention group, characterized with the daily consumption of almonds, showed increased vitamin E concentrations by 102.7%, reductions in total cholesterol (5.5%) and LDL-C (6.4%) levels, and other important markers (IL-10, ICAM-1, IL-1β, IL-6) in comparison with the cookie group. These results suggested that including almonds in the daily diet could improve nutritional status and reduce the risk of cardiovascular diseases [83]. Other authors have confirmed that almonds could have a beneficial effect on cardiovascular health status. Choudhury et al. (2014) concluded that an almond-enriched diet, consisting of eating 50 g almonds/day for 4 weeks, could increase plasma α-tocopherol and enhance vascular function by decreasing systolic blood pressure and improving low-mediated dilatation [84].

Regarding peanuts, Caldas et al. (2020) performed a clinical trial with 64 overweight men who were randomly allocated in three groups: the control (following a diet that was nut-free), conventional peanuts consumers (56 g/day), and high-oleic peanut groups (56 g/day). After 4 weeks of intervention, the activity of glutathione S transferase and superoxide dismutase remained unchanged in both peanut groups. Further human intervention studies should be carried out to elucidate the potential benefits of consuming peanuts in terms of different oxidative markers [85].

Among all nuts, current scientific evidence has suggested that pistachio nuts generally contain the highest amounts of gamma-tocopherol as well as different bioactive compounds with an interesting antioxidant activity such as beta-carotene, lutein, and zeaxanthin [86]. Various clinical trials have been focused on studying the effects of pistachio nuts on certain oxidative stress parameters. Kay et al. (2010) and Gulati et al. (2014) conducted randomized control trials in humans who were randomly allocated either in intervention groups (consumption of pistachio nuts) or in a control group. The results obtained in these studies demonstrated a significant reduction in the serum oxidized LDL levels [87,88]. In addition, Kay et al. (2010) reported higher plasma concentrations of lutein, α-carotene, and β-carotene (bioactive compounds with important antioxidant properties) in those subjects who followed a pistachio-enriched diet [89]. Gulati et al. (2014) observed a marked increase in the plasma antioxidant activity, expressed as the capacity to inhibit thiobarbituric acid reactive substances (TBARSs), in the intervention group; however, long-term trials are necessary to confirm these findings [87]. In addition, a reduction in total oxidative status and higher activity of superoxide dismutase were found in a prospective study carried out by Sari et al. (2010) with 32 healthy young men. In the intervention group, individuals were instructed to follow the Mediterranean Diet with a high consumption of pistachio nuts for 4 weeks. In comparison with the control group, improvements in some indices of oxidation and lipid peroxidation, such as lipid hydroperoxide and malondialdehyde levels, were shown with a diet rich in pistachio nuts [88]. A recent randomized crossover clinical trial performed by Canudas et al. (2019) with 49 prediabetic individuals revealed that a pistachio-supplemented diet decreased DNA damage caused by oxidative stress [90].

As previously indicated, oxidative stress is an important factor involved in the development of several pathologies, including cardiovascular diseases or neurodegenerative disorders. Food habits directly impact different biomarkers related to oxidative stress and inflammation, and so, adherence to a healthy dietary pattern is essential to maintain an adequate health status. In this sense, the antioxidant and anti-inflammatory properties of the Mediterranean Diet have been widely investigated. Participants in a long-term intervention with a Mediterranean Diet enriched with extra virgin olive oil, carried out by Martinez-Lapiscina et al. (2013), showed significant improvements in cognitive function in comparison with adults subjected to a control diet [91]. Another study, conducted in 2014 by Carnevale et al., suggested that the inclusion of extra virgin olive oil in a Mediterranean-type diet is aimed to protect against postprandial oxidative stress [92]. In this line, it was observed that Mediterranean Diet, rich in high quality extra virgin olive oil, induced an increase in the number of lactic acid bacteria in gut microbiota that could reduce inflammation and oxidative stress in normal and overweight/obese subjects [93]. The effect of the daily consumption of extra virgin olive oil and nuts, within the context of a Mediterranean Diet, was studied by Casas et al. (2014), who selected 164 participants at high risk for cardiovascular diseases. The results revealed that the intervention had both had an important anti-inflammatory effect as well as led to an improvement in the cardiovascular status of the participants [94]. Recently, Estruch et al. (2018) performed, in Spain, a multicenter trial that enrolled 7447 participants with high cardiovascular risk. Individuals were grouped in three clusters according to three different diets: a Mediterranean diet supplemented with extra virgin olive oil, a Mediterranean diet supplemented with mixed nuts, and a control diet (advice to reduce dietary fat). The authors observed that a Mediterranean diet supplemented with extra virgin olive oil or nuts was associated with a lower incidence of major cardiovascular events such as stroke, myocardial infarction, and even death from cardiovascular causes [95].

To the authors’ knowledge, few studies have been conducted to assess the antioxidant properties potentially shown by date fruits (*Phoenix dactylifera* L.). As an example, Rock et al. (2009) carried out a pilot study with healthy participants who consumed 100 g/day of two different varieties of date fruits (Medjool and Hallawi varieties) for 4 weeks. According to the results obtained, the basal serum oxidative status was significantly reduced by 33% in those individuals consuming the Hallawi variety in comparison with the levels observed before date fruit consumption [96].

Finally, few scientific studies have been performed with rye, an ingredient commonly used in food production. Söderholm et al. (2012) designed a dietary trial with 63 healthy females to clarify the potential benefits provided by a high consumption of rye bread within the context of a varied and balanced diet. A significant improvement in the oxidation resistance of LDL particles was shown after the rye bread intervention; however, more clinical trials are necessary to clarify these preliminary results [97].

**Figure 2:**
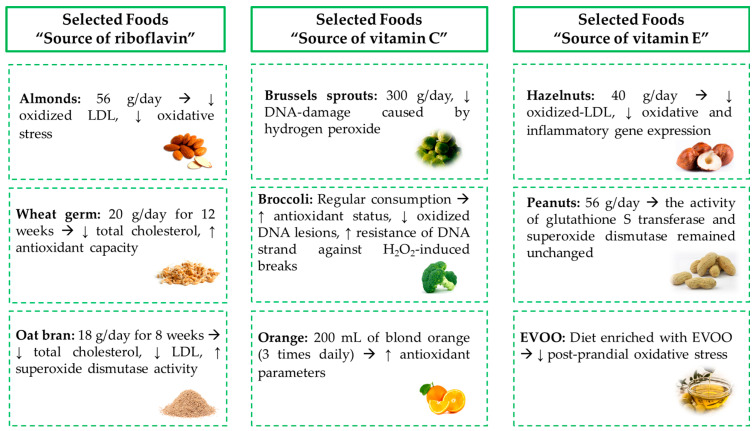
Findings of consumption of some selected foods that are sources of riboflavin, vitamin C, and vitamin E and the oxidative stress prevention. Based on [49,51,54,67,68,69,70,72,80,81,85,92].

Figure 2 graphically represents some findings reported in the manuscript related to consumption of some selected foods that are sources of riboflavin, vitamin C, and vitamin E and the oxidative stress prevention.

## 3. Materials and Methods

Firstly, a detailed review of the vitamins with health claims related to oxidative stress prevention, approved in the European Union (EU), was performed using the public information available on the official website of the EC, concretely, the European Register of nutrition and health claims made for food and food supplements (https://ec.europa.eu/food/safety/labelling_nutrition/claims/register/public/?event=register.home, accessed on 21 November 2022). Database filters applied to focus the search were the following: “claims status: authorized”, “type of claim: Art. 13”, “EFSA Opinion reference: all”, and “Legislation: all” [32].

Secondly, the selection of foods of plant origin subjected to this review was performed according to two criteria: they had (1) to be natural sources of the vitamin in question, in compliance with the European regulations in force (Regulation (EC) 1924/2006 and Regulation (EU) 1169/2011); and (2) to have the highest contents of the vitamin in question, in accordance with data published in the Official Databases of food composition BEDCA “https://www.bedca.net/ (accessed on 6 June 2023)”, FRIDA “https://frida.fooddata.dk/?lang=en (accessed on 6 June 2023)”, and USDA “https://fdc.nal.usda.gov/ (accessed on 6 June 2023).

Thirdly, a literature review was carried out in different scientific databases and resources such as PubMed “https://pubmed.ncbi.nlm.nih.gov/ (accessed on 30 June 2023)”, Science Direct “https://www.sciencedirect.com/ (accessed on 30 June 2023)”, and Google Scholar “https://scholar.google.es/ (accessed on 30 June 2023)”. Scientific opinions published by the EFSA on its official website “https://www.efsa.europa.eu/en/publications?s=&page=2 (accessed on 3 July 2023)” were considered as well.

Lastly, clinical trials conducted in humans were selected among the studies with the highest levels of scientific evidence. English language, year of publication, and the keywords ‘vitamin’, ‘antioxidant’, ‘oxidative stress’, ‘dietary sources’, and ‘foods of plant origin’ were defined as the inclusion criteria as well. Both scientific studies that directly focused on other bioactive compounds, different from the ones selected in the present review, as well as trials performed in animals and/or in vitro studies were not considered (exclusion criteria). In addition, studies assessing the effect of supplementation with pharmaceutical forms (capsules, powder, etc.) were not selected.

## 4. Conclusions

The search for new strategies to reduce oxidative processes in humans is a remarkable research line within the scientific community. Most of these strategies include the study of functional foods as promising tools to reduce the risks of the appearance of certain chronic diseases and pathologies.

Riboflavin, vitamin C, and vitamin E are micronutrients with important properties related to oxidative stress prevention, and they are subject to one health claim referring to the protection of DNA, proteins, and lipids from oxidative damage. Several scientific studies related to natural food sources of riboflavin (almonds, wheat germ, mushrooms, and oat bran), vitamin C (guava, kale, black currant, Brussels sprouts, broccoli, and orange), and vitamin E (hazelnuts, almonds, peanuts, pistachio nuts, extra virgin olive oil, dates, and rye) have been performed and published in the literature. However, no food of plant origin has obtained a favorable EFSA opinion to substantiate the approval of health claims related to its potential antioxidant properties.

Further studies (concretely, well-controlled human intervention studies) must be carried out in accordance with EFSA requirements to provide the highest level of scientific evidence that could demonstrate the potential relationship between foods of plant origin and antioxidant capacity. The present review could be useful for the scientific community to study the application of health claims referring to the antioxidant capacity potentially exerted by foods of plant origin.

## Figures and Tables

**Table 1:** Use conditions applied to the health claim, related to the protection of DNA, proteins, and lipids from oxidative damage, approved for riboflavin (vitamin B_2_), vitamin C, and vitamin E [28,38,39].

Vitamins	Health Claim	Use Conditions of the Health Claim (“Source of”)		
		NRV	Beverages	Foods
Riboflavin (vitamin B2)	“Contributes to the protection of cells from oxidative stress”	1.4 mg	≥0.105 mg/100 mL	≥0.21 mg/100 g
Vitamin C		80 mg	≥6 mg/100 mL	≥12 mg/100 g
Vitamin E		12 mg	≥0.9 mg/100 mL	≥1.8 mg/100 g

NRV = Nutrient Reference Value.

**Table 2:** Examples of foods of plant origin that are sources of riboflavin [40,41,42].

Food	Riboflavin Content (mg/100 g Edible Portion)
Almond	0.78–1.14
Wheat germ	0.5–0.61
Wheat bran	0.36–0.6
Mushroom	0.35–0.41
Kale	0.29–0.35
Soy flour	0.28–1.16
Rice bran	0.28
Lupin	0.22
Oat bran	0.22
Pinto bean	0.21–0.22

**Table 3:** Examples of foods of plant origin that are sources of vitamin C [40,41,42].

Food	Vitamin C Content (mg/100 g Edible Portion)
Guava	176–228
Kale	169–93.4
Black currant	159.6–181
Brussels sprouts	140–85
Red pepper	128–163
Broccoli	89.2–117
Cauliflower	76.8–48.2
Papaya	60.9–78.5
Kiwi	59–74.7
Orange	50–54.4

**Table 4:** Examples of foods of plant origin that are sources of vitamin E [40,41,42].

Food	Vitamin E Content (mg/100 g Edible Portion)
Hazelnut	15–26
Almond	23.4–25.6
Wheat germ	11–21
Extra virgin olive oil	18
Peanut	6.6–10.9
Pistachio nut	2.9–5.2
Avocado	1.9–3.2
Date	2.2
Soy flour	2–2.2
Rye	1.9

## Data Availability

Data sharing is not applicable to this article.
